# Condition-dependent effects of Elexacaftor/Tezacaftor/Ivacaftor (Trikafta) on *Aspergillus fumigatus* growth

**DOI:** 10.1128/spectrum.02275-24

**Published:** 2025-07-30

**Authors:** Elise Biquand, Sandra Khau, Delphine Fouquenet, Samanta Chouela Mbouwe, Floriane Costes, Lucile Drobecq, Adélaïde Chesnay, Guillaume Desoubeaux, Benoit Briard

**Affiliations:** 1 Centre d’Étude des Pathologies Respiratoires, Inserm UMR1100, Université de Tours, Tours, France; 2 Parasitologie-Mycologie médicale-Médecine tropicale, Pôle Biologie médicale-Pathologie, hôpital Bretonneau, Tours, France; Universidade do Minho, Braga, Portugal

**Keywords:** *Aspergillus fumigatus*, cystic fibrosis, cftr modulators, Elexakaftor/Tezacaftor/Ivacaftor

## Abstract

Fungal infection, especially allergic bronchopulmonary aspergillosis, is a leading cause of infection-associated morbidity and death in patients with cystic fibrosis. We have previously discovered that the new leading treatment in cystic fibrosis, CFTR modulators Elexacaftor/Tezacaftor/Ivacaftor (ETI, Trikafta), drastically reduces the colonization and infection by *Aspergillus fumigatus*. However, the reasons for this decrease in patients are not known so far. In this study, we have shown, using *A. fumigatus* reference strains and strains isolated from patients with cystic fibrosis, that CFTR modulators have no discernible impact on initial conidia growth *in vitro*. However, in a condition-dependent manner, we demonstrated a decrease in the minimal effective concentration (MEC) of caspofungin when administered with ETI on conidia. By contrast, during macrophage infection, we observed that high concentrations of ETI treatment promoted fungal growth but inhibited inflammasome activation.

**IMPORTANCE:**

The advent of ETI therapy represents a pivotal moment, signaling the onset of major changes in the medical field of cystic fibrosis and its related infectious diseases. However, the impact of ETI treatment on the patient’s microbiota and pathogens has to be further studied as proof arises of changes in patient colonization.

## OBSERVATION

We previously published pioneering data that underscored a substantial change in the impact of *Aspergillus*-related diseases in patients with cystic fibrosis (pwCF) treated with a combination of cystic fibrosis transmembrane conductance regulator (CFTR) modulators, so-called Trikafta (1). Our results highlighted a noticeable reduction in the proportion of respiratory samples colonized by *Aspergillus* spp. In addition, there was a statistically significant decrease in anti-*Aspergillus* precipitin reactivity and total IgE concentration following the initiation of Trikafta treatment. These findings suggested a robust decline in fungal development and colonization. Several mechanisms could be raised to explain such outcomes. For instance, Elexakaftor/Tezacaftor/Ivacaftor (ETI, Trikafta) could suppress the overall inflammatory response. Indeed, by reducing neutrophil activation, moderating the release of reactive oxygen species (ROS) and cytokines, and restoring macrophage-induced phagocytosis (2), the therapeutic combination can make the local environment more challenging for microbial colonization and development of immune hyperreactivity. Furthermore, next-generation sequencing (NGS) revealed that CFTR modulators enhance microbial diversity in respiratory samples, restoring the steady state of the microbiota: major colonizer pathogens being less represented, while minor species regained their prominence (3). For instance, lung colonization by *Pseudomonas aeruginosa* bacillus was shown to be decreased by 24.2% in 124 patients receiving ETI for 1 year (4).

However, the precise mechanism of action of CFTR modulators remains elusive in such a context. It is acknowledged that some non-anti-infective drugs can *in vitro* exert a direct antimicrobial effect against pathogens: for example, mycophenolate mofetil—conventionally used for the prevention of graft rejection—presents low minimal inhibitory concentrations (MICs) against various fungal species (5) and might play a critical role in *in vivo* strain selection (6).

To investigate this, we assessed fungal growth from the conidial stage of two reference strains (DAL and Af293) and four clinical isolates (two issued from ETI-treated patients and two from non-treated pwCF), in different conditions with or without ETI treatment. Several media were exploited for the *in vitro* cultures: Dulbecco’s modified Eagle medium (DMEM), Roswell Park Memorial Institute medium (RPMI), as well as minimal medium (MM), which is a defined medium classically used to test compounds acting on *A. fumigatus*. ETI drugs were tested at two different concentrations: serum concentrations ([3VX]_S_) based on clinical observations from therapeutic drug monitoring in treated patients (9.5 µM VX-445 [Elexacaftor], 6.53 µM VX-661 [Tezacaftor], and 2.29 µM VX-770 [Ivacaftor]) (7); and effective concentrations ([3VX]_E_) established according to *in vitro* efficacy data on epithelial cells to restore CFTR channel activity (3 µM VX-445, 18 µM VX-661, and 1 µM VX-770) (8).

**Fig 1:**
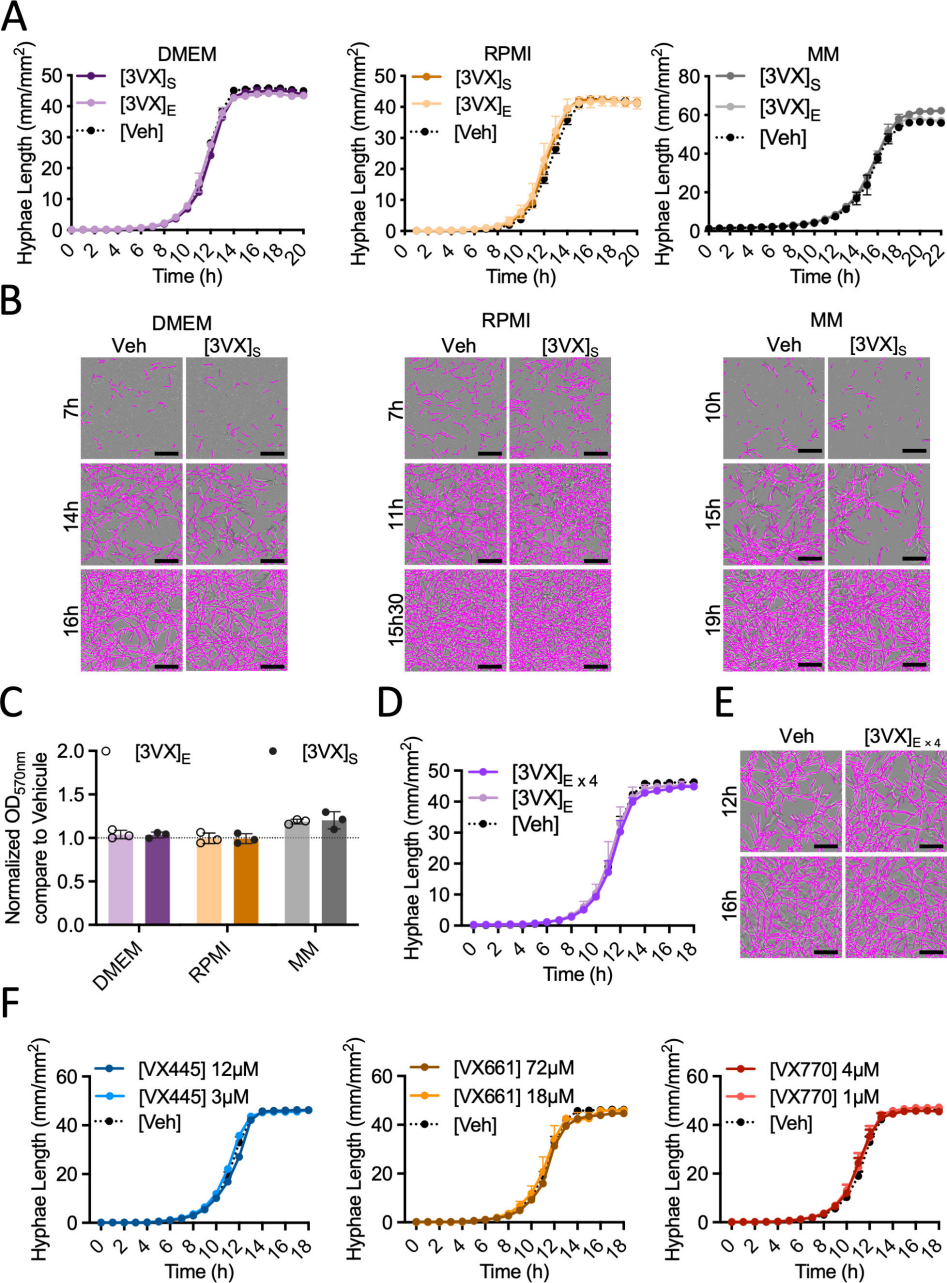
Evaluation of the direct antimicrobial effects of CFTR modulators (Elexacaftor, VX445/Tezacaftor, VX661/Ivacaftor, VX770) on *Aspergillus fumigatus* growth and biofilm formation. (**A**) Measurement of *A. fumigatus* hyphal length (Strain: DAL) using the IncuCyte live-cell analysis system in DMEM and RPMI media supplemented with 10% fetal bovine serum (FBS) or minimal medium (MM) in the presence of 3VX (VX445/VX661/VX770) treatment at effective concentration ([3VX]_E_) or the serum concentration found in treated cystic fibrosis patients ([3VX]_S_), as well as with vehicle ([Veh]; DMSO). The shown data depict a representative experiment; the experiment was independently replicated at least three times, with measurements taken hourly in each well. (**B**) Representative images of *A. fumigatus* (Strain: DAL) biofilm at indicated times and media with or without 3VX treatment at patient serum concentration. Fungal hyphae were detected using the neurotrack module (Sartorius) and highlighted in purple. Scale bars, 200 µm. (**C**) Assessment of biofilm formation (Strain: DAL) by crystal violet testing and endpoint measurement at 570 nm after culture in DMEM and RPMI media supplemented with 10% FBS or MM (*n* = 3 for each condition). (**D**) Measurement of hyphal length of *A. fumigatus* (Strain: DAL) in DMEM with 10% FBS, with or without 3VX treatment at effective concentration or fourfold effective concentration. (**E**) Representative images of *A. fumigatus* (Strain: DAL) biofilm at 12 and 16 h in DMEM with or without 3VX treatment at a fourfold effective concentration. Scale bars, 200 µm. (**F**) Measurement of hyphal length of *A. fumigatus* (Strain: DAL) in DMEM with 10% FBS with VX445, VX661, or VX770 at the effective concentration or fourfold effective concentration. Data are shown as mean ± SD. Representative experiments are shown for A, B, and D through F.

Regardless of the combination of concentrations ([3VX]_S_ and [3VX]_E_) or the medium used, the initial growth of *A. fumigatus* DAL strain was not perturbed by ETI treatment (Fig. 1A and B). Similarly, fungal biofilm development remained unaffected by the treatment (Fig. 1C; Fig. S1). Interestingly, none of the clinical strains obtained from pwCF who were either treated or untreated with ETI exhibited susceptibility to the treatment compared to the vehicle condition (Fig. S2). These results suggest that ETI treatment does not affect conidial growth or biofilm establishment when conidia are exposed to the treatment from the outset.

Furthermore, despite a significant increase (fourfold) in the combined concentration of all three ETIs, no effects were observed on the initial growth of *A. fumigatus* (Fig. 1D and E). We wondered whether the three compounds would exhibit activity when used separately on *A. fumigatus*. We tested each compound at two different concentrations, corresponding to the effective concentration or a fourfold increase. However, at all concentrations tested, the initial growth *of A. fumigatus* was not affected by any of the compounds compared to the vehicle condition (Fig. 1F).

Considering our results, it is plausible to assert that the ETI combination does not exert a direct antimicrobial or stimulatory effect *in vitro* on *A. fumigatus* conidia when exposed to actual *in vivo* ETI concentrations. Interestingly, Jones *et al*. (9) recently showed that on a preformed biofilm that at 5 or 10 µM CFTR modulators were able to dramatically reduce fungal biomass by 50%, increase cell permeability close to amphotericin B activity, and raise metabolic activity of *A. fumigatus* by 20%–30% (9). Therefore, we decided to increase the compound concentration to 10 µM for each and test the effect on resting conidia growth in RPMI, DMEM, or MM media. Unfortunately, no significant effect was observed on initial fungal growth compared to the vehicle control (Fig. S3). However, one should note that this previous work was performed on the preformed hyphal stage, which may explain the difference (9). Indeed, we observed that *A. fumigatus* biofilm maturation decreased when the ETI treatment was added to pre-established biofilm; however, the effect was lower with the serum and effective concentration (Fig. S4). As patients present lower serum concentrations of modulators and are infected by conidia, the effect of ETI treatment on *A. fumigatus* growth is presumably indirect. As it has been shown that ETI treatment sensitizes *Aspergillus* to antifungal drugs (9), we assessed whether the fungus is more sensitive to caspofungin and voriconazole when treated. Incubation of conidia with ETI treatment does not alter the minimal inhibitory concentration of voriconazole (data not shown). However, in the presence of ETI, the minimal effective concentration (MEC) of caspofungin was reduced (Fig. S5). These findings align with the observations of Jones *et al.,* who reported that ETI treatment enhances the effect of caspofungin in pre-established biofilm. However, our data suggest that the improvement in caspofungin efficacy is independent of any additional inhibitory effect of ETI on *A. fumigatus* biofilm formation. Instead, the increased sensitivity of *A. fumigatus* to caspofungin may result from ETI’s impact on plasma membrane permeability, which could enhance the accessibility of caspofungin to its target, β−1,3-glucan synthase. Such increased sensitivity could participate in the observed reduction of *Aspergillus* infections in patients (1).

**Fig 2:**
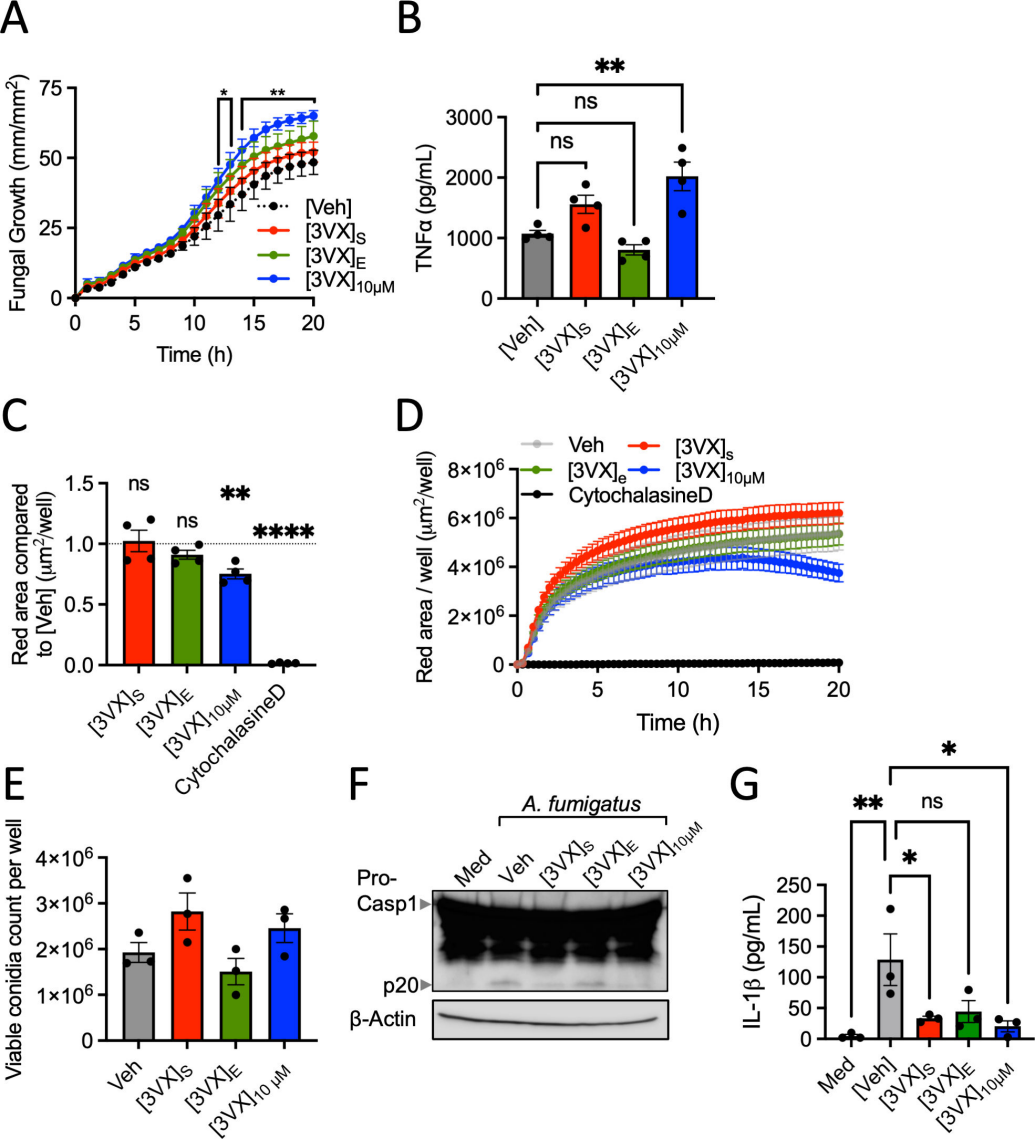
High concentration of CFTR modulators (Elexacaftor [VX445], Tezacaftor [VX661], and Ivacaftor [VX770]) impairs macrophage control of *A. fumigatus* growth. (**A**) Measurement of *A. fumigatus* fungal length (Strain: DAL-DSred) infecting bone marrow-derived macrophages (BMDM) at a multiplicity of infection (MOI) of 5, using the IncuCyte live-cell analysis system in the presence of 3VX (VX445/VX661/VX770) treatment at [3VX]_S_, [3VX]_E_, or [3VX]_10μM_, as well as with [Veh]. *P < 0.05, **P < 0.01 (two-way ANOVA with Dunnett’s multiple comparisons test); data are presented as mean ± SEM, *n* = 3. (**B**) Release of TNF⍺ in BMDMs was assessed after 20 h with *A. fumigatus* (MOI of 15). **P < 0.01 (one-way ANOVA with Dunnett’s multiple comparisons test); data are presented as mean ± SEM, *n* = 4. (**C**) Normalized phagocytosis to vehicle control ([Veh]) at 20 h time point. **P < 0.01, ****P < 0.005 (one sample t test, theoretical mean of 1); data are presented as mean ± SEM, *n* = 4. (**D**) Representative measurement of zymosan phagocytosis by primed BMDMs in the presence of 3VX treatment at concentrations [3VX]_S_, [3VX]_E_ or [3VX]_10μM_, as well as in the presence of cytochalasine D or [Veh]. (**E**) Quantification of viable conidia following 4 h of BMDM infection in the presence of 3VX treatment at [3VX]_S_, [3VX]_E_, or [3VX]_10μM_, as well as with [Veh]. (**F**) Immunoblot analysis of pro-caspase-1 (**P45**), the caspase-1 subunit p20 (**P20**), and β-actin of primed BMDMs left untreated (medium alone [Med]) or in the presence of 3VX treatment at [3VX]_S_, [3VX]_E_, or [3VX]_10μM_, as well as with [Veh] during 20 h after infection with *A. fumigatus* (MOI of 15). (**G**) Release of IL-1β in BMDMs assessed after 20 h infection with *A. fumigatus* (MOI of 15) left untreated (medium alone [Med]) or in the presence of 3VX treatment at [3VX]_S_, [3VX]_E_, or [3VX]_10μM_, as well as with [Veh]. *P < 0.05, **P < 0.01 (one-way ANOVA with Dunnett’s multiple comparisons test); data are presented as mean ± SEM, *n* = 3.

However, when we tested the initial growth of *A. fumigatus* conidia in the presence of macrophages, we observed that high concentrations of ETI treatment facilitated fungal growth during the early stages (Fig. 2). Interestingly, similar results were observed with *Cftr^ΔF508/ΔF508^* macrophages, which appeared even more sensitive to the treatment (Fig. S6). Alongside the increased fungal growth, we noted an elevated release of the pro-inflammatory cytokine TNF⍺ (Fig. 2B).

This finding contrasts with the observations made by Jones *et al.*, where TNF⍺ levels were reported to decrease (9). However, this discrepancy is likely due to differences in the cell types tested between the two studies—macrophages in our study *versus* hematopoietic stem cells in theirs. These results suggest that ETI treatment impairs macrophage control of *A. fumigatus* growth and simultaneously amplifies inflammation. A similar observation was made in macrophages treated with ETI and infected with *Pseudomonas aeruginosa*, where ETI treatment also failed to reduce inflammatory cytokine levels (10).

To understand how the ETI treatment may affect the control of *A. fumigatus* growth by macrophages, we evaluate the impact of ETI on macrophage phagocytic activity and phagolysosome maturation. We observed that phagocytosis and phagolysosome maturation were reduced in the presence of a high dose of ETI ([3VX]_10μM_) compared to the vehicle control ([Veh]), whereas no significant effect was observed at lower concentrations ([3VX]_S_ and [3VX]_E_) (Fig. 2C and D). Since phagocytic activity directly influences the ability of macrophages to kill conidia, we next assessed the impact of ETI treatment on macrophage-mediated killing of resting conidia. However, no significant changes were observed, regardless of the ETI concentration used (Fig. 2E).

To further explore the mechanism by which ETI treatment influences *A. fumigatus* control during bone marrow-derived macrophage (BMDM) infection, we examined its impact on inflammasome activation. The inflammasome is a pro-inflammatory complex that mediates the activation and release of pro-inflammatory cytokines, which is crucial in controlling *A. fumigatus* infection (11, 12). Interestingly, ETI treatment alters mitochondrial activity in macrophages. Specifically, it inhibits baseline oxygen consumption in both CF and non-CF monocyte-derived macrophages, without affecting glycolytic activity (10). Given the established link between metabolic activity and inflammasome activation (13), we hypothesized that ETI treatment may alter inflammasome activation and impair the host’s ability to control the fungus. We observed that ETI treatment directly affects caspase-1 cleavage and IL-1β production at the concentration of [3VX]_S_ and [3VX]_10μM_, while the difference at [3VX]_E_ was not statistically significant (Fig. 2F and G). This observation confirmed that the treatment perturbed the immune response of the host cells to pathogens and may explain the altered control of the fungal growth. Interestingly, a similar alteration in inflammasome activation was observed in *Cftr*^Δ^*^F508/^*^Δ^*^F508^* BMDMs (Fig. S6).

Overall, our results demonstrate that ETI treatment exerts pleiotropic, context-dependent effects on *A. fumigatus*. When administered during the initial growth phase *of A. fumigatus*, ETI does not significantly alter fungal growth, except in the presence of caspofungin, where antifungal activity is enhanced. By contrast, when applied to pre-established biofilms, ETI impacts both biofilm maturation and the activity of caspofungin. These findings may explain the observed decrease in fungal isolation in patients newly treated with ETI. However, patients remain exposed to inhaled conidia, which directly interact with the treatment. We propose that the most significant factor in the reduction of *A. fumigatus* colonization is the marked improvement in respiratory capacity and mucus clearance associated with Trikafta therapy.

In addition, we found that *A. fumigatus* growth and inflammatory cytokines such as TNF⍺ release were elevated in macrophages treated with ETI. By contrast, the inflammasome-dependent cytokine IL-1β was reduced, along with an impairment in inflammasome activation. Notably, ETI was shown to inhibit mitochondrial function in macrophages, a process essential for controlling fungal growth and promoting antifungal immunity, which may explain these results (14, 15). This observation warrants further investigation and careful consideration, as it raises the possibility that patients undergoing Trikafta treatment might develop renewed susceptibility to *A. fumigatus* infections over time.

Thus, the introduction of ETI therapy marks a crucial milestone, heralding the start of significant transformations in the medical landscape for cystic fibrosis and related infectious diseases, but more mechanistic investigations on the modulation of microbiota and mycobiota are still needed.

### *Aspergillus* strains and culture

Two *Aspergillus fumigatus* reference strains were used, DAL (CEA10, CBS144.89) and Af293 (NCPF7367), and four clinical isolates (SAfT14 & SAfT16 issued from ETI-treated patients, and SAfT13 & SAfT15 from non-treated patients). *A. fumigatus* strains were grown on Sabouraud dextrose agar slants for 1  week. Conidia were harvested in water containing 0.1% (vol/vol) Tween-20 and filtered through a cell strainer 40 µM (Fisherbrand). *In vitro* cultures were realized in three media: Dulbecco’s modified Eagle medium (DMEM, Gibco) and Roswell Park Memorial Institute medium (RPMI, Gibco), both supplemented with 10% fetal bovine serum (FBS), as well as minimal medium (MM) (16). ETI drugs (Ivacaftor (VX-770), Tezacaftor (VX-661), and Elexacaftor (VX-445) (MedChemExpress, HY-13017, HY-15448 & HY-111772)) were mixed and tested at two different concentrations: serum concentrations ([3VX]_S_ = 9.5 µM VX-445, 6.53 µM VX-661, and 2.29 µM VX-770); and effective concentrations ([3VX]_E_ = 3 µM VX-445, 18 µM VX-661, and 1 µM VX-770). Vehicle suspension (DMSO) was used as a negative control.

### Growth assay

The level of fungal growth of 1.10^3^ *A. fumigatus* conidia, deposited in wells of a 96-well plate, was continuously monitored at 37°C by an IncuCyte live-cell analysis system (Sartorius, Göttingen—Germany) for 20 h in DMEM + 10% FBS and RPMI +10% FBS, or 24 h for MM. Hyphae length was analyzed using the NeuroTrack module of IncuCyte (17). At the endpoint, the biofilm was stained with 0.01% crystal violet, washed two times, and discolored with 30% acetic acid, to measure absorbance at 570 nm with the Multiskan Sky spectrophotometer (Thermo Fisher Scientific, Waltham, MA—USA) (18). The total biomass of *A. fumigatus* biofilm was estimated by measuring the fungal dry weight as follows: in a 24-well plate, conidia were seeded in MM and treated with ETI at the corresponding concentration for 40 hours at 37°C. Alternatively, conidia were allowed to grow for 16 hours before the addition of ETI at the corresponding concentration, followed by an additional 24 hour incubation at 37°C. The mycelium was then collected and centrifuged at 10,000 × *g* for 10 minutes. After removing the supernatant, the pellet was frozen, lyophilized, and weighed to determine the dry weight.

### Bone marrow-derived macrophage isolation and infection

Bone marrow cells were harvested from the femurs and tibias of mice C57BL6/Jand CFTR^ΔF508/ΔF508 (19) by flushing the bones with sterile phosphate-buffered saline (PBS). The resulting cell suspension was passed through a 70 µm cell strainer to remove debris and centrifuged at 400 × *g* for 5 minutes. After discarding the supernatant, red blood cells were lysed using NaCl solutions. The remaining cells were resuspended and cultured in 20 cm Petri dishes (639161, Greiner) containing BMDM differentiation medium, composed of RPMI 1640 with GlutaMAX supplement (Gibco, 61870-010), 15% macrophage colony-stimulating factor (M-CSF) conditioned medium from NIH 3T3 cells, 10% fetal bovine serum (FBS), 1% penicillin/streptomycin, 1% non-essential amino acids (NEAA), and 1× β-mercaptoethanol. Cultures were maintained at 37°C in an incubator with 5% CO₂ for 6 days. On day 6, differentiated BMDMs were seeded into 24-well cell culture plates (353047, Falcon) at a density of 5 × 10⁵ cells per well. The following day, cells were primed with 100 ng/mL lipopolysaccharide (LPS) (L9143, Sigma-Aldrich) and 50 ng/mL interferon-gamma (IFNγ) (P01579, Invivogen) for 5 hours. BMDMs were subsequently infected with *Aspergillus fumigatus* conidia at a multiplicity of infection (MOI) of 5 or 15 for 4 hours or 16 hours.

### Conidia survival assay

After 4 hours of incubation with activated BMDM, supernatant was removed, and BMDM was lysed with 0.1% Triton X100 solution for 5 minutes at room temperature. Serial dilutions of the suspension were then spread on Sabouraud agar plates and incubated overnight at 35°C before counting.

### Cytokine analysis

Cytokine levels were determined by ELISA using kits according to the manufacturer’s instructions: TNF⍺ DuoSet ELISA (DY410, R&D Systems) and IL-1β (DY401, R&D Systems).

### Zymosan phagocytosis assay

BMDM cells were plated at 8.10^4^ cells/well in a 96-well plate and primed. Cells were then treated with 5 µg of pHrodo zymosan Bioparticles for Incucyte (Sartorius), and fluorescence was monitored for 20 h in the Incucyte (Sartorius).

### Western blots

Lysis buffer (5× buffer: 10% NP40, 10 mM DTT, antiproteases cocktail (11697498001, Roche) was directly added to the supernatant along with 4× loading dye, and samples were denatured at 95°C for 10 min before loading on a 13% polyacrylamide gel. After transfer and blocking in TBS-Tween 20 0.1% + milk 5%, membranes were incubated overnight at 4°C with either an anti-Caspase1 antibody (Adipogen, Ag-20B-0042-C100, dilution: 1/1,000) or an anti-Actin-HRP (Sigma, A3854, dilution: 1/5,000). Reveal was done using the ECL Clarity substrate for HRP (Biorad, #1705060).

## SUPPLEMENTAL MATERIAL

Fig. S1 spectrum.02275-24-s0001.tiff Evaluation of the direct antimicrobial effects of CFTR modulators on *Aspergillus fumigatus* conidia growth and biofilm formation by monitoring the fungal biomass.

Fig. S2 spectrum.02275-24-s0002.tiff Evaluation of the direct antimicrobial effects of CFTR modulators on *Aspergillus fumigatus* DAL strain.

Fig. S3 spectrum.02275-24-s0003.tiff Evaluation of the effect of conidia concentration on the antimicrobial effects of CFTR modulators on pre-established *Aspergillus fumigatus* biofilm.

Fig. S4 spectrum.02275-24-s0004.pdf The CFTR modulators impair CftrΔF508/ΔF508 macrophage control of *Aspergillus fumigatus* growth.

Fig. S5 spectrum.02275-24-s0005.pdf CFTR modulators (Elexacaftor [VX445], Tezacaftor [VX661], and Ivacaftor [VX770]) enhance the activity of Caspofungin against *A. fumigatus*.

Fig. S6 spectrum.02275-24-s0006.pdf The CFTR modulators impair CftrΔF508/ΔF508 macrophage control of *A. fumigatus* growth.

ASM does not own the copyrights to Supplemental Material that may be linked to, or accessed through, an article. The authors have granted ASM a non-exclusive, world-wide license to publish the Supplemental Material files. Please contact the corresponding author directly for reuse.
