# Supplementary figures and images for: Condition-dependent effects of Elexacaftor/Tezacaftor/Ivacaftor (Trikafta) on Aspergillus fumigatus growth
Source: Microbiol Spectr. 2025 Jul 30;13(9):e02275-24. doi: 10.1128/spectrum.02275-24 (PMC12403852; doi:10.1128/spectrum.02275-24)

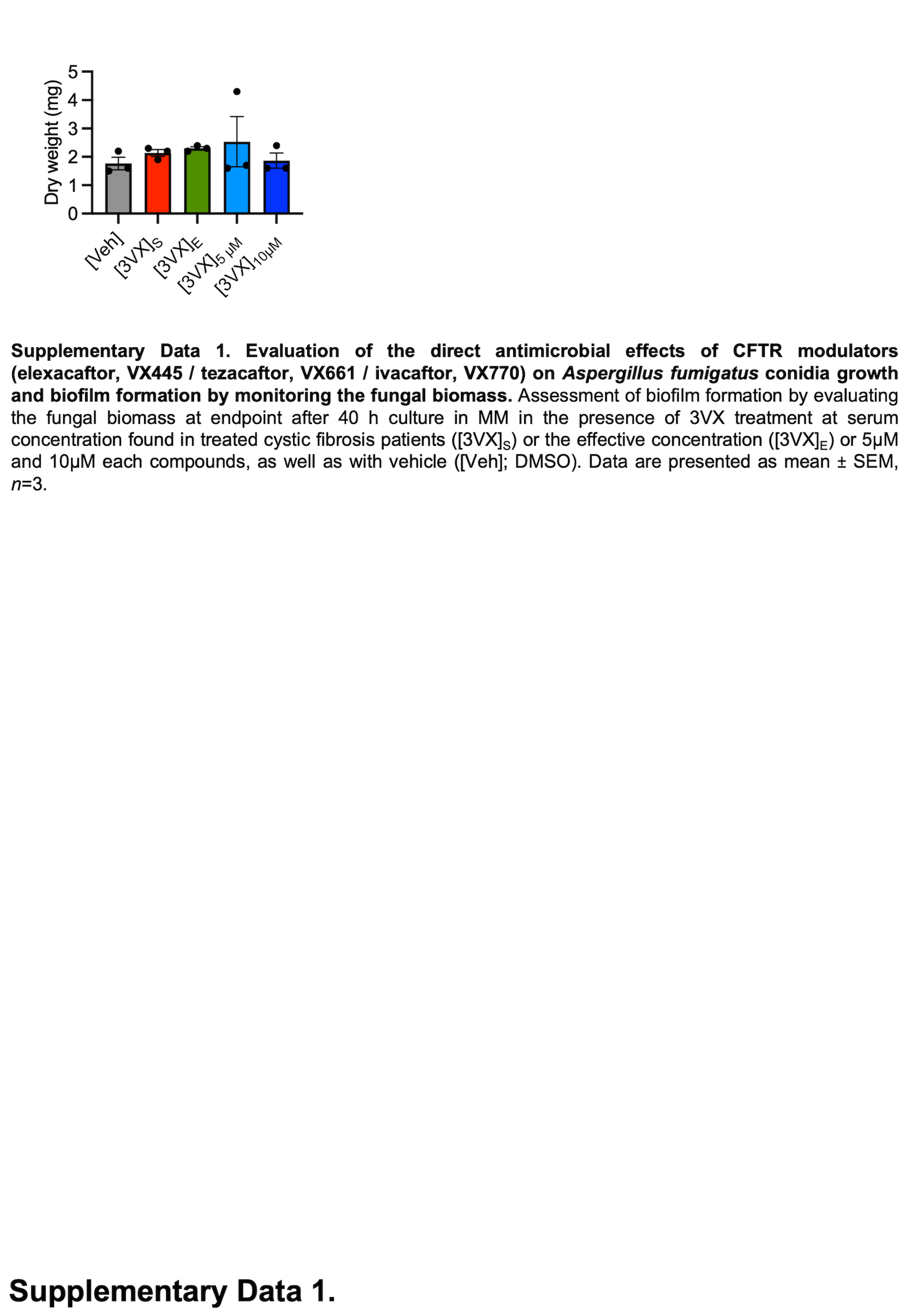

Supplement: Fig. S1 — Evaluation of the direct antimicrobial effects of CFTR modulators on Aspergillus fumigatus conidia growth and biofilm formation by monitoring the fungal biomass. [file spectrum.02275-24-s0001.tiff]

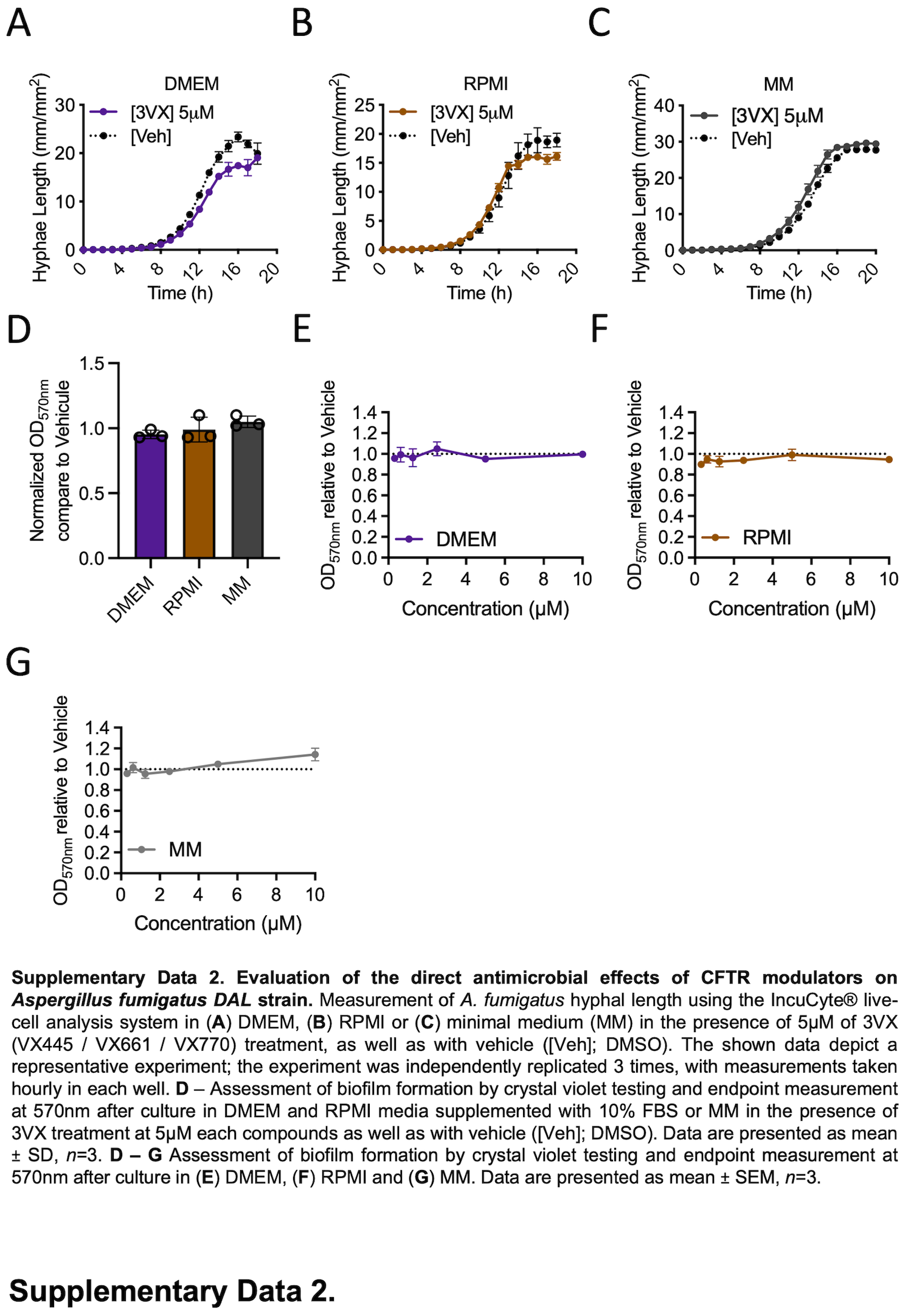

Supplement: Fig. S2 — Evaluation of the direct antimicrobial effects of CFTR modulators on Aspergillus fumigatus DAL strain. [file spectrum.02275-24-s0002.tiff]

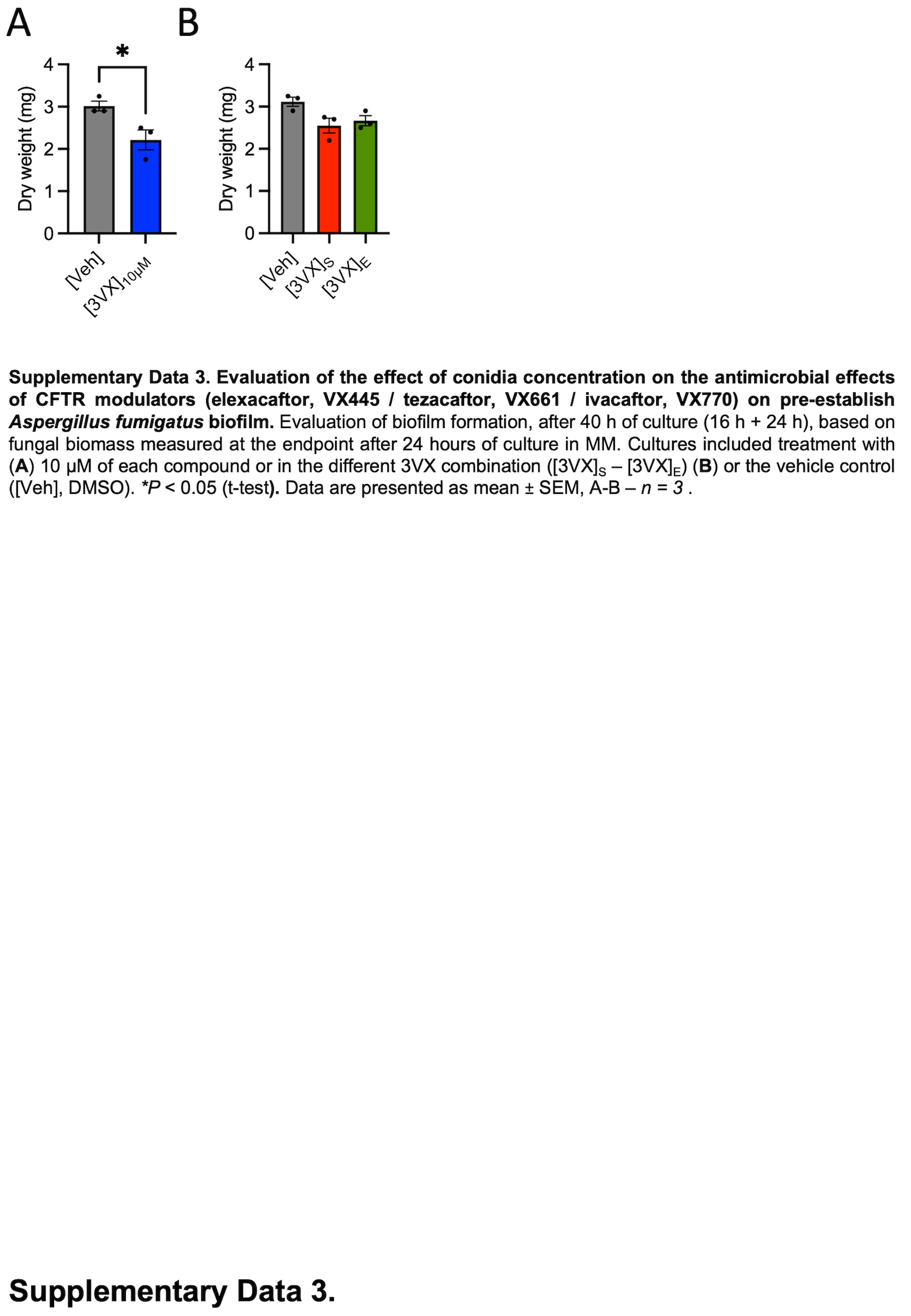

Supplement: Fig. S3 — Evaluation of the effect of conidia concentration on the antimicrobial effects of CFTR modulators on pre-established Aspergillus fumigatus biofilm. [file spectrum.02275-24-s0003.tiff]
