# Supplementary material for: Condition-dependent effects of Elexacaftor/Tezacaftor/Ivacaftor (Trikafta) on Aspergillus fumigatus growth
Source: Microbiol Spectr. 2025 Jul 30;13(9):e02275-24. doi: 10.1128/spectrum.02275-24 (PMC12403852; doi:10.1128/spectrum.02275-24)
Supplement: Fig. S4 — The CFTR modulators impair CftrΔF508/ΔF508 macrophage control of Aspergillus fumigatus growth. [file spectrum.02275-24-s0004.pdf]

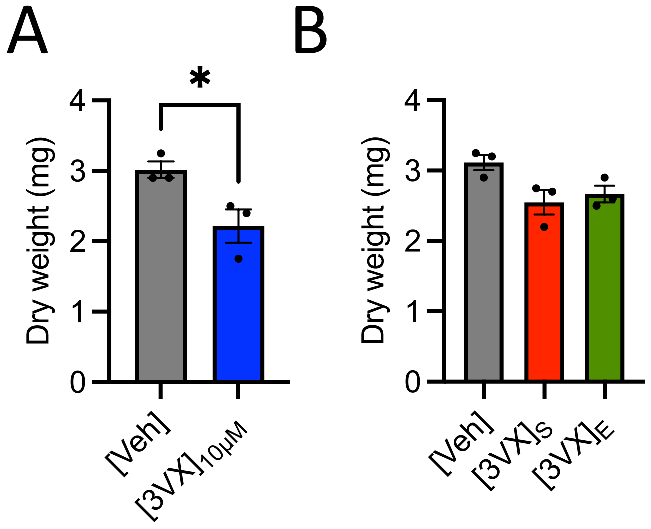

Fig. S4. Evaluation of the effect of conidia concentration on the antimicrobial effects of CFTR modulators (Elexacaftor, VX445 / Tezacaftor, VX661 / Ivacaftor, VX770) on pre-establish *Aspergillus fumigatus* biofilm. Evaluation of biofilm formation, after 40 h of culture (16 h + 24 h), based on fungal biomass measured at the endpoint after 24 hours of culture in MM. Cultures included treatment with (**A**) 10 µM of each compound or (**B**) in the different 3VX combination ($[3VX]_S$ – $[3VX]_E$) or the vehicle control ([Veh], DMSO). $^*P < 0.05$ (t-test**).** Data are presented as mean ± SEM, A-B – $n = 3$.

Fig. S4
