# Supplementary material for: Condition-dependent effects of Elexacaftor/Tezacaftor/Ivacaftor (Trikafta) on Aspergillus fumigatus growth
Source: Microbiol Spectr. 2025 Jul 30;13(9):e02275-24. doi: 10.1128/spectrum.02275-24 (PMC12403852; doi:10.1128/spectrum.02275-24)
Supplement: Fig. S5 — CFTR modulators (Elexacaftor [VX445], Tezacaftor [VX661], and Ivacaftor [VX770]) enhance the activity of Caspofungin against A. fumigatus. [file spectrum.02275-24-s0005.pdf]

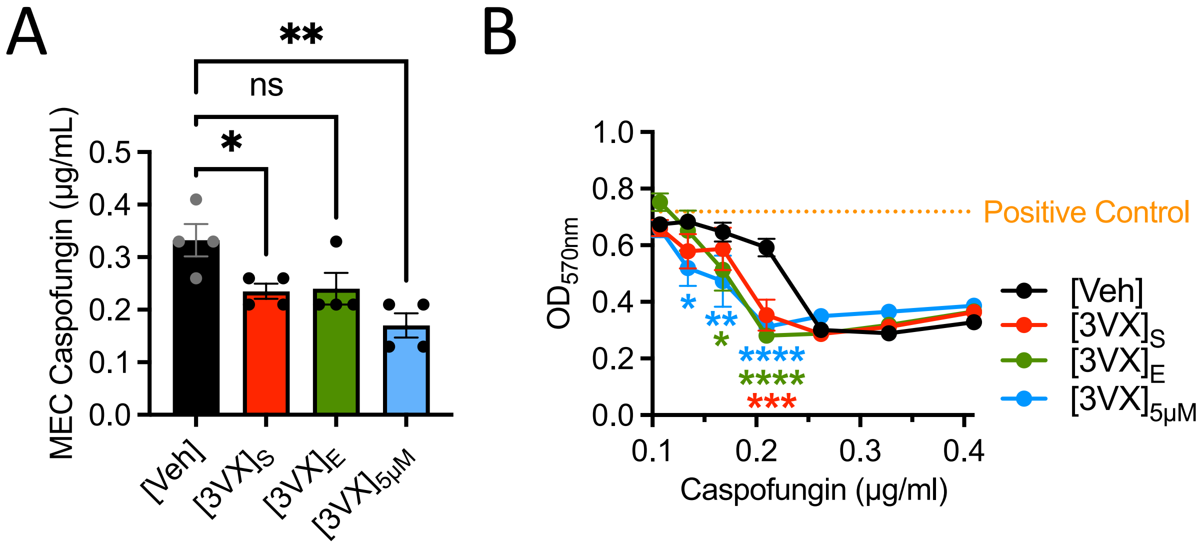

Fig. S5. CFTR modulators (Elexacaftor [VX445], Tezacaftor [VX661], and Ivacaftor [VX770]) enhance the activity of Caspofungin against *A. fumigatus*. A – Evaluation of the Minimal Effective Concentration (MEC) of Caspofungin on *A. fumigatus* (Strain: DAL-DSred) conidia growth in MM. Cultures included treatment at serum concentration of 3VX found in treated cystic fibrosis patients ($[3VX]_S$) or the effective concentration ($[3VX]_E$) or 5 µM of each compounds, as well as with vehicle ([Veh]; DMSO). **B** – Assessment of biofilm formation by crystal violet testing and endpoint measurement at optical density (OD) of 570nm after 28 h of culture of resting conidia in MM in the presence of 3VX treatment $[3VX]_S$, $[3VX]_E$, $[3VX]_{5µM}$ or [Veh]. Non treated conidia represent positive control. Data are presented as mean ± SEM, *n* = 4.

Fig. S5
