# Supplementary material for: Condition-dependent effects of Elexacaftor/Tezacaftor/Ivacaftor (Trikafta) on Aspergillus fumigatus growth
Source: Microbiol Spectr. 2025 Jul 30;13(9):e02275-24. doi: 10.1128/spectrum.02275-24 (PMC12403852; doi:10.1128/spectrum.02275-24)
Supplement: Fig. S6 — The CFTR modulators impair CftrΔF508/ΔF508 macrophage control of A. fumigatus growth. [file spectrum.02275-24-s0006.pdf]

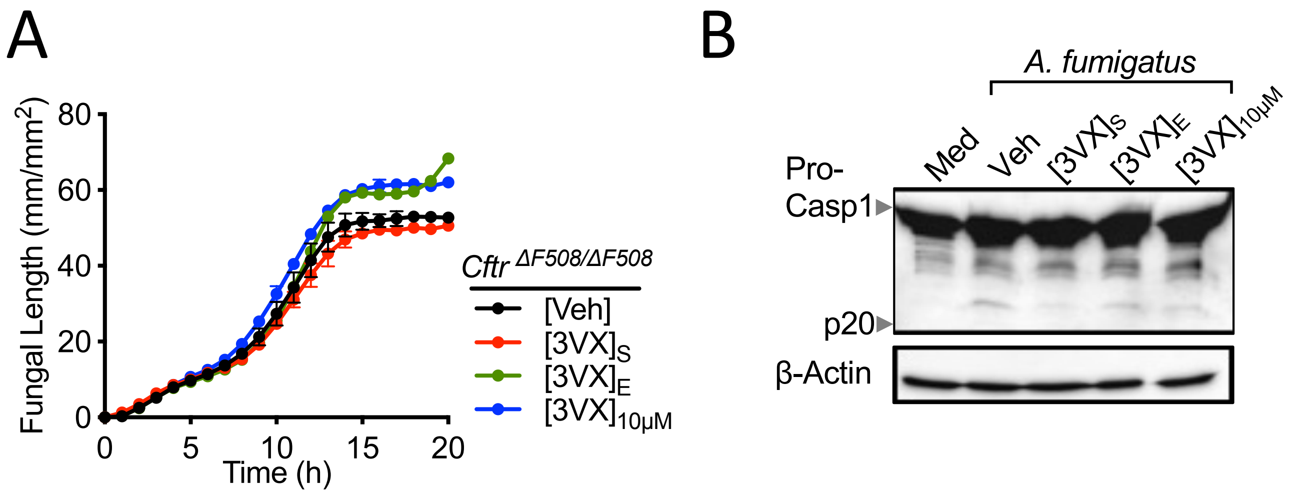

Fig. S6. The CFTR modulators impair *Cftr*$^{\Delta F508/\Delta F508}$ macrophage control of *A. fumigatus* growth. A – Measurement of *A. fumigatus* fungal length (Strain: DAL-DSred) infecting *Cftr*$^{\Delta F508/\Delta F5}$ BMDM at MOI of 5, using the IncuCyte® live-cell analysis system in the presence of 3VX (VX445 / VX661 / VX770) treatment at $[3VX]_S$, $[3VX]_E$ or $[3VX]_{10\mu M}$, as well as with [Veh]. *n* = 1. **B** – Immunoblot analysis of pro-caspase-1 (p45), the caspase-1 subunit p20 (p20) and β-actin of primed BMDMs left untreated (medium alone (Med)) or in the presence of 3VX (VX445 / VX661 / VX770) treatment at $[3VX]_S$, $[3VX]_E$ or $[3VX]_{10\mu M}$, as well as with [Veh] during 20 h after infection with live *A. fumigatus* (MOI of 15). *n* = 1.

Fig. S6
